# Failure to Detect the Novel Retrovirus XMRV in Chronic Fatigue Syndrome

**DOI:** 10.1371/journal.pone.0008519

**Published:** 2010-01-06

**Authors:** Otto Erlwein, Steve Kaye, Myra O. McClure, Jonathan Weber, Gillian Wills, David Collier, Simon Wessely, Anthony Cleare

**Affiliations:** 1 Jefferiss Research Trust Laboratories, Section of Infectious Diseases, Wright-Fleming Institute, Faculty of Medicine, Imperial College London, St Mary's Campus, Norfolk Place, London, United Kingdom; 2 Social Genetic and Developmental Psychiatry Centre, Institute of Psychiatry (King's College London) De Crespigny Park, Denmark Hill, London, United Kingdom; 3 Department of Psychological Medicine, Institute of Psychiatry, King's College London, Camberwell, London, United Kingdom; University of California San Francisco, United States of America

## Abstract

**Background:**

In October 2009 it was reported that 68 of 101 patients with chronic fatigue syndrome (CFS) in the US were infected with a novel gamma retrovirus, xenotropic murine leukaemia virus-related virus (XMRV), a virus previously linked to prostate cancer. This finding, if confirmed, would have a profound effect on the understanding and treatment of an incapacitating disease affecting millions worldwide. We have investigated CFS sufferers in the UK to determine if they are carriers of XMRV.

**Methodology:**

Patients in our CFS cohort had undergone medical screening to exclude detectable organic illness and met the CDC criteria for CFS. DNA extracted from blood samples of 186 CFS patients were screened for XMRV provirus and for the closely related murine leukaemia virus by nested PCR using specific oligonucleotide primers. To control for the integrity of the DNA, the cellular beta-globin gene was amplified. Negative controls (water) and a positive control (XMRV infectious molecular clone DNA) were included. While the beta-globin gene was amplified in all 186 samples, neither XMRV nor MLV sequences were detected.

**Conclusion:**

XMRV or MLV sequences were not amplified from DNA originating from CFS patients in the UK. Although we found no evidence that XMRV is associated with CFS in the UK, this may be a result of population differences between North America and Europe regarding the general prevalence of XMRV infection, and might also explain the fact that two US groups found XMRV in prostate cancer tissue, while two European studies did not.

## Introduction

A recent study by Lombardi *et al.* [1] describing a gamma-retrovirus infection in 68 of 101 chronic fatigue syndrome (CFS) patients was notable not only for its claim of a new viral aetiology of a hitherto controversial disease, but also for the fact that proviral DNA could be amplified from the peripheral blood mononuclear cells (PBMC) of 3.75% (8/218) of the healthy controls. This follows an earlier claim that 1.7% (5/300) of healthy Japanese blood donors carried antibodies to the same virus [2]. The virus in question is a recently discovered retrovirus, Xenotropic Murine Leukaemia Virus (MLV)-Related Virus (XMRV).

In the original identification of XMRV in prostate cancer stromal cells, Urisman *et al.* [3] confirmed by sequence analysis that XMRV is not a laboratory contaminant, as is often the case with claims of new retroviral associations with disease. It shares >90% sequence identity in *gag* and *env* (two of the three viral structural genes) with other xenotropic MLVs.

An association between XMRV and prostate cancer was strengthened with the demonstration of XMRV protein expression in malignant epithelial cells [4]. However, these results have not been duplicated in studies conducted in Europe [5]–[7]. Both prostate cancer and CFS have been linked to an Arg to Gln mutation at codon 462 (R462Q) in the RNaseL gene, an interferon-induced ribonuclease [8]. On activation, RNaseL destroys single stranded cellular and viral RNA, thereby preventing viral replication, blocking protein synthesis, triggering cellular apoptosis and providing an innate anti-viral response. The two US studies are of interest, not only because this would be a further example of a virus association with cancer, but because they represent the first demonstration of a gamma-retrovirus able to infect human cells, over-riding the intrinsic immune mechanisms that were believed to protect humans from MLV infection.

The XMRV sequences derived from prostate cancer tissue are identical to those from CFS patients, but differ from xenotropic MLV sequences, endorsing a genuine cross-species transmission. However, the claim that XMRV is preferentially found in prostate tumours from patients homozygous for the R462Q variant [3] is not borne out by the second prostate cancer study to find XMRV in patients [4], nor was the genetic variant detected in CFS patients carrying XMRV [5].

The finding of Lombardi *et al.* of a 67% XMRV infection rate among CFS patients, if confirmed, would have a serious impact on understanding the pathogenesis of this complex and debilitating disease and its treatment. Therefore, it was important to determine if CFS sufferers in the UK were carriers of XMRV. We have screened DNA extracts from the blood of CFS sufferers by PCRs targeted at an XMRV-specific sequence and at a sequence conserved amongst most murine retroviruses (MRV).

## Methods

### Patients

All patients gave written informed consent for the use of their DNA to test aetiological theories of CFS, and the study was approved by the South London and Maudsley NHS Trust Ethics Committee. The study recruited 186 patients (62% female, age range 19–70, mean 39.6±11.3years) from consecutive referrals to the CFS clinic at King's College Hospital, London. All patients had undergone medical screening to exclude detectable organic illness, including a minimum of physical examination, urinalysis, full blood count, urea and electrolytes, thyroid function tests, liver function tests, 9 a.m. cortisol and ESR. Patients were interviewed using a semi-structured interview for CFS [9] to determine whether they met international consensus criteria for CFS. All subjects met the CDC criteria [10]; patients with the Fukuda-specified exclusionary psychiatric disorders, or somatisation disorder (as per DSM-IV), were not included. The patient set studied is a well-characterised and representative sample of CFS patients who have been described previously: all were routine clinic attendees, referred within the UK National Health Service, who had taken part in prior studies of neuroendocrine functioning [11] and/or of cognitive behaviour therapy [12]. As is typical of the patients seen in this tertiary care centre, they were markedly unwell. Few were working, and 19% were members of patient support groups for CFS/ME [12]–[14]. The levels of fatigue in this sample were high (mean Chalder Fatigue Scale, 26.3±5.4) [15], as were levels of disability (mean Work and Social Adjustment Scale, total score 28.2±7.2) [16]. The mean GHQ-12 score [17] was 19.7±8.1. Patients had been unwell for a median of 4.0 y (range 1–28 y). Of note was that 45% said their illness definitely related to a viral illness and 45% said it might relate to a viral illness. Overall, we conclude that this sample is typical of CFS patients seen in specialist clinical services in the UK. We also know from collaborative studies that our patients resemble those seen in other specialist CFS services in the United States and Australia [18].

#### PCR detection of XMRV and MLV sequences

DNA was extracted from EDTA whole blood using a standard phenol-based organic deproteinisation procedure [19]. DNA concentrations were determined by absorbance at 260 nm (A_260_). Each sample was amplified in three nested PCRs using primers targeted to an XMRV-specific sequence, to a sequence conserved amongst most MLV and, as a control for sample addition and PCR-inhibition, to a human beta-globin (hBG) sequence (Table 1). Each first-round reaction was performed in a 25 µl volume containing 0.5 units TaqGold (Applied BioSystems, Warrington, UK), 1 x TaqGold reaction buffer (Applied BioSystems), 1.5 mM Mg^2+^, 200 mM each dNTP, 2.5 pmol each primer to which 5 µl DNA extract or control was added. Reaction conditions were one cycle of 94°C, 8 minutes, 35 cycles of 94°C 30 seconds, 55°C 30 seconds, 72°C 30 seconds and one cycle 0f 72°C, 7 minutes. Second round reaction mixes were identical to the first round and the sample was a 1 µl transfer from the first round reactions. Second round reaction conditions were as for the first round over 30 cycles. PCR amplicons were visualised on a 1% agarose gel stained with ethidium bromide. Each PCR run consisted of test samples, six negative (water) and two positive controls. The positive control was a dilution of a plasmid with a full-length XMRV (isolate VP62) insert, generously gifted by Dr R. Silverman. To validate the sensitivity of the PCR, an end-point dilution of the plasmid was performed. To determine specificity of the PCR, a sample of human DNA from the LNCaP prostate cancer cell line (American Type Culture Collection, code CRL-1740) was amplified with the XMRV and MLV primer sets. To ensure integrity of the DNA extracts, three randomly selected samples were titrated to end-point using the hBG PCR to determine if the PCR copy number equated with the A_260_. To determine if the DNA extracts exhibited low level non-specific inhibition of PCR, 10 samples were subjected to 30 cycles of the first round hBG PCR (reaction mix and conditions as above) followed by 40 cycles of a nested real-time SYBR-green PCR using the SYBR-green Fast PCR kit (Roche, Lewes UK) according to the manufacturer's instructions.

**Table 1:** Oligonucleotide Primers.

Target	Sequence		Location
**XMRV**	Forward outer	5′CATTCTGTATCAGTTAACCTAC 3′	411–432^1^
	Reverse outer	5′ ATGATCTCGAGAACACTTAAAG 3′	606–588^1^
	Forward inner	5′ GACTTTTTGGAGTGGCTTTGT 3′	441–461^1^
	Reverse inner	5′ ACAGAAGAACAACAAAACAAATC 3′	566–544^1^
**MLV**	Forward outer	5′ GGATCAAGCCCCACATACAG 3′	2796–2847^1^
	Reverse outer	5′ CATCAAACAGGGTGGGACTG 3′	3179–3160^1^
	Forward inner	5′ AGAAGTCAACAAGCGGGTGG 3′	2926–2945^1^
	Reverse inner	5′ GGTGGAGTCTCAGGCAGAAA 3′	3062–3043^1^
**hBG**	Forward outer	5′ TGGTGGTCTACCCTTGGACC 3′	148–162^2^
	Reverse outer	5′ GAGGTTGTCCAGGTGAGCCA 3′	296–277^2^
	Forward inner	5′ GAGGTTCTTTGAGTCCTTTGG 3′	170–190^2^
	Reverse inner	5′ CATCACTAAAGGCACCGAGCA 3′	273–253^2^

Locations in GenBank accessions ^1^EF185282, ^2^NM000518.4.

## Results

### Nested PCR Validation

Based on A_260_ of the purified plasmid, both primer sets (XMRV, MLV) were able to amplify a single target copy added to the reaction. Amplification of 600 ng of LNCaP cellular DNA added to XMRV and MLV PCRs yielded no non-specific bands when viewed on an ethidium bromide-stained agarose gel. Quantification of DNA samples from three randomly selected test samples by end-point dilution PCR with the hBG primer set showed concurrence of the PCR-determined copy number with A_260_, thus indicating integrity of the DNA preparations. Nested real-time amplification of 10 samples showed no evidence of non-specific inhibition as determined by the slope of the amplification curves and the height of the signal plateau.

### PCR Analysis of Test Samples

**Figure 1:**
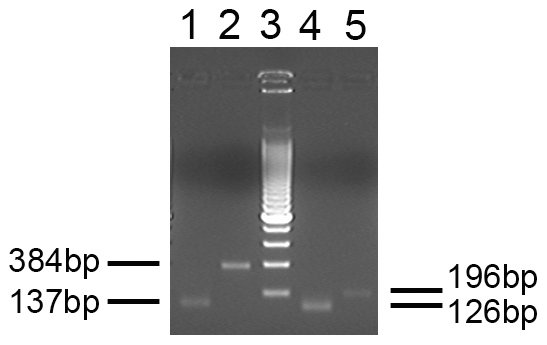
PCR products of the XMRV VP62 clone. Primers are generic to MLV (lanes 1 and 2) or specific to XMRV (lanes 4 and 5). The sizes of the respective fragments are shown. Lane 3–200 bp molecular size ladder.

**Figure 2:**
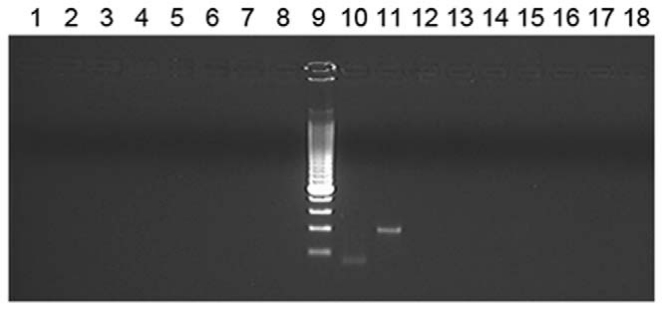
Nested PCR from the DNA of 8 CFS patients. Products of generic MLV primers (including XMRV) are shown. Lanes 1–8, CFS patient DNA (2^nd^ round); lanes 9 and 10, XMRV 2^nd^ round and 1^st^ round positive controls; lanes 11 and 12, DNA of uninfected cell line LNCaP; lanes 13–18, water controls.

Input DNA ranged from 10 to 600 ng (1.6×10^3^ to 1.1×10^5^ cell equivalents) as determined by A_260_ of which 149 samples had an input of >100 ng and 106 samples >200 ng. None of the 186 test samples analysed yielded a specific PCR product with either the XMRV or MLV primer sets and no non-specific PCR products were observed. A specific hBG product was amplified from all 186 test samples. The positive control was amplified in each run by the XMRV and MLV primer sets. A stained gel of the XMRV and MLV PCR products is shown in figure 1 and a representative sample of our results with CFS DNA and MLV primers is shown in figure 2.

## Discussion

Unlike the study of Lombardi *et al*., we have failed to detect XMRV or closely related MRV proviral DNA sequences in any sample from CFS cases. There have been numerous claims for an infective aetiology to CFS over the years, not least because, as in this sample, many patients report that their symptoms were triggered by an infective episode. Prospective epidemiological studies have confirmed that certain infective agents, for example Epstein Barr virus, are unequivocally associated with subsequent CFS [20], even if the mechanisms are unclear and almost certainly multi factorial. Nearly two decades ago, sequences from another retrovirus, the human T-lymphotropic virus type ll, were amplified from the PBMCs of 10/12 (83%) adult and 13/18 paediatric CFS patients, but not from healthy control subjects [21]. However, subsequent studies carried out on small numbers (20–30) of CFS patients, failed to confirm evidence for HTLV (type 1 or 11) [22]–[25] or other retroviruses, including the closely-related simian T lymphotropic virus type l, the prototype foamy virus, simian retrovirus, bovine and feline leukaemia viruses [26] and HIV-1 [23].

The Lombardi paper is the first to study a significantly larger number of people than that in any previous study and to detect a virus only recently discovered. Our study resembles that of Lombardi *et al.* in certain respects. Both studies use the widely accepted 1994 clinical case definition of CFS^10^. Lombardi *et al*. reported that their cases “presented with severe disability” and we provide quantifiable evidence confirming high levels of disability in our subjects. Our subjects were also typical of those seen in secondary and tertiary care in other centres.

Our own study also differs from that of Lombardi in other respects. Firstly, the PCR operator was blinded to the provenance of the DNA samples. In fact, with the exception of the PCR controls, all 186 DNA test samples originated from CFS patients. Care was taken to grow the XMRV plasmid in a laboratory in which no MLV had been cultured and no MLV vectors used and the PCR was carried out in a CPA-accredited Molecular Diagnostics Unit which processes only human tissue. Multiple (six) water (negative) controls were included in every run to detect low level contamination and a PCR to amplify a sequence that is conserved in most murine leukaemia viruses was included in order to expose any circulating MLV contamination and to detect any variant of XMRV that might be circulating in the UK CFS population.

Based on our molecular data, we do not share the conviction that XMRV may be a contributory factor in the pathogenesis of CFS, at least in the U.K.
